# Diffusion-Weighted Magnetic Resonance Imaging in Ovarian Cancer: Exploiting Strengths and Understanding Limitations

**DOI:** 10.3390/jcm11061524

**Published:** 2022-03-10

**Authors:** Tanja Gagliardi, Margaret Adejolu, Nandita M. deSouza

**Affiliations:** 1 Department of Imaging, The Royal Marsden NHS Foundation Trust, London SW3 6JJ, UK; tanja.gagliardi@rmh.nhs.uk (T.G.); margaret.adejolu@rmh.nhs.uk (M.A.); 2 Division of Radiotherapy and Imaging, The Institute of Cancer Research, London SW7 3RP, UK

**Keywords:** diffusion-weighted MRI, high grade serous ovarian cancer, quantitation, apparent diffusion coefficient, tumor characterization, tumor staging, treatment response

## Abstract

Detection, characterization, staging, and response assessment are key steps in the imaging pathway of ovarian cancer. The most common type, high grade serous ovarian cancer, often presents late, so that accurate disease staging and response assessment are required through imaging in order to improve patient management. Currently, computerized tomography (CT) is the most common method for these tasks, but due to its poor soft-tissue contrast, it is unable to quantify early response within lesions before shrinkage is observed by size criteria. Therefore, quantifiable techniques, such as diffusion-weighted magnetic resonance imaging (DW-MRI), which generates high contrast between tumor and healthy tissue, are increasingly being explored. This article discusses the basis of diffusion-weighted contrast and the technical issues that must be addressed in order to achieve optimal implementation and robust quantifiable diffusion-weighted metrics in the abdomen and pelvis. The role of DW-MRI in characterizing adnexal masses in order to distinguish benign from malignant disease, and to differentiate borderline from frankly invasive malignancy is discussed, emphasizing the importance of morphological imaging over diffusion-weighted metrics in this regard. Its key role in disease staging and predicting resectability in comparison to CT is addressed, including its valuable use as a biomarker for following response within individual lesions, where early changes in the apparent diffusion coefficient in peritoneal metastases may be detected. Finally, the task of implementing DW-MRI into clinical trials in order to validate this biomarker for clinical use are discussed, along with the trials that include it within their protocols.

## 1. Introduction

High grade serous ovarian cancer (HGSOC) is the leading cause of death from gynecologic malignancy (age-adjusted mortality rates 12.5/100,000 women in the UK, [1] and 6.3/100,000 women in the USA [2]). It typically presents late with non-specific symptoms of abdominal pain and bloating in post-menopausal women—80% of cases are diagnosed in women over 50 years of age. An important determinant of ovarian cancer survival is the stage of disease at diagnosis. Five-year survival rates are greater than 90% for Stage I disease (disease confined to the ovaries), but fall very sharply to <10% for late stage cases [1]. The majority (60%) of women are diagnosed with stage III (widespread dissemination through the abdomen and pelvis) or IV disease (disease extends to the pleural cavity or involves mediastinal, cardiophrenic, or sometimes inguinal nodes, or an umbilical nodule), and only 30% are diagnosed at the earliest stage [1].

HGSOC is an epithelial tumor. Where disease is localized to the pelvis, distinction from benign (endometriomas, mature teratomas, thecomas, and cystadenomas), borderline or other malignant (immature teratomas and granulosa cell tumors) ovarian masses is essential to establish the correct management pathway. Standard management involves primary debulking (cytoreductive) surgery (PDS) followed by adjuvant taxane–platinum combination chemotherapy [3]. Suboptimal debulking surgery negatively impacts prognosis [4,5,6], while complete cytoreduction has a positive effect. Two landmark phase III clinical trials (European Organization for Research and Treatment of Cancer [EORTC] 55,971 and primary chemotherapy versus primary surgery for newly diagnosed advanced ovarian cancer [CHORUS trial]) show that neoadjuvant chemotherapy (NACT) followed by interval debulking surgery (IDS) is a suitable alternative to standard PDS followed by chemotherapy, and presents an opportunity to increase the number of women who could benefit from complete cytoreductive surgery [7,8]. Although randomized clinical trial data show no clear superiority for either management option [5], it is well established that completeness of surgical resection, regardless of its timing, is the strongest predictor of disease-free survival [9,10]. Patients who undergo surgery which results in inadequate removal of visible disease (disease left behind >1 cm in diameter) gain little or no benefit but endure significant side-effects and complications from such surgery [11] and are better managed with a policy of chemotherapy from the outset. Selecting the optimal timing of surgery also reduces the length of surgical procedures (operating time), post-surgical complications and length of hospital stays. Therefore, the best possible pre-operative imaging to characterize the tumor and inform the extent of surgery required and its timing is essential to enable the best possible patient outcome. Additionally, in recurrent ovarian cancer, preoperative imaging also plays a major role in assessing surgical resectability because incomplete secondary cytoreductive surgery in these cases may be of little value in improving overall survival [6,12,13].

Making a surgical decision on resectability relies heavily on imaging assessment of disease extent and location. To date, contrast enhanced computerized tomography (CT) scans of the abdomen and pelvis have formed the mainstay of this imaging assessment and are considered standard-of-care as CT is easily available and the imaging modality is robust and relatively cheap. However, even when contrast enhanced, CT lacks the soft-tissue contrast to detect disease at potentially “difficult-to-resect” sites. The superior soft tissue contrast of T2-W magnetic resonance imaging (MRI) together with techniques such as diffusion-weighted (DW-) MRI provide coverage of the abdomen and pelvis with high contrast between tumor and non-tumor tissue [14,15] and, therefore, may be increasingly used for delineating the full extent and location of tumor deposits in the abdomen and pelvis. This article focuses on the role specifically of DW-MRI in detecting, staging, and following-up ovarian cancer and its current role as a quantitative biomarker in clinical trials.

## 2. Optimizing the DW-MRI Technique

In DW-MRI, the MR signal is sensitized to motion by the inclusion of two additional magnetic field gradients within the pulse sequence [16]. The first gradient pulse alters the phase shift of each proton by an amount dependent on the water molecule’s spatial location relative to the gradient. The second gradient pulse (equal and opposite in effect to the first) reverses this phase shift if the water molecule does not move between the application of the first and second gradient pulses. Brownian motion, however, causes a characteristic mean displacement between gradient pulses, leading to an imperfect reversal of the phase and, hence, a loss of phase-coherence among spins. This process manifests itself as signal loss in the macroscopic image voxel giving rise to an image whose contrast is determined by diffusive processes [17]. The degree of signal loss is directly proportional to the degree of water motion, which, in turn, is dependent on the protons’ mean diffusional path lengths. Through thermal diffusion of water in tissues, pathological processes that result in changes in cell volume, membrane integrity, or modulations of the extracellular matrix can, therefore, be probed since the effective diffusion length depends on the spatial details of the structures restricting their motion. Pure water at body temperature with a self-diffusion coefficient of D~3 × 10^−3^ mm^2^/s and evolution time of ~50 ms has a root-mean-square displacement of ~30 µm, which means that in cells (dimension ~1–15 µm), water molecules will encounter many cellular and subcellular impediments.

**Figure 1:**
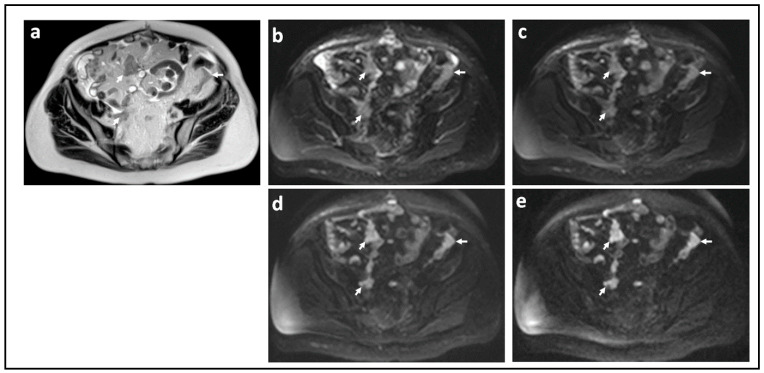
Peritoneal metastases in high grade serous ovarian cancer showing increasing lesion conspicuity with increasing *b*-value: T2-W (**a**) and DW-MRI at *b*-values of 0 s/mm^2^ (**b**), 100 s/mm^2^ (**c**), 500 s/mm^2^ (**d**), and 900 s/mm^2^ (**e**). The irregular metastatic deposits on the surface of the bowel and in the mesentery (arrows) show diffusion restriction. They increase in conspicuity and contrast as diffusion weighting increases because they retain signal while signal from adjacent normal tissues diffuses away.

The sensitivity of the DW-MRI sequence to diffusion is characterized by its *b*-value (a combination of gradient pulse amplitude, the time for which the gradients are applied, and the time that elapses between their application). These parameters can be adjusted to alter the sensitivity of the sequence to diffusion. The higher the *b*-value, the more sensitive an image is to the effects of diffusion. An optimal high *b*-value to image peritoneal metastases from ovarian cancer is around 1000 s/mm^2^ [18] (Figure 1). Microcirculation of blood in the capillary bed (perfusion) means that perfusion contributes to the loss in signal on a DW-MRI image. Fortunately, the movement of water due to microcirculation has a diffusivity 10 times larger than true diffusion [19] and are normally evident when *b*-values are below 100 s/mm^2^ [20]. A range of *b*-values between 100 and 1000 s/mm^2^ are ideal for delineating metastatic ovarian cancer [21].

DW-MRI is quantifiable. The apparent diffusion coefficient (ADC) of each voxel can be calculated in square millimeters per second automatically by current MR systems by assuming a monoexponential model of signal decay between two or more *b* values. The ADC represents the slope of the curve between the natural logarithm of measured signal intensity for different *b* values. More robust ADC calculations are obtained when multiple *b* values and multiexponential models are used [22], which may be more accurate. A low ADC signifies a short average diffusional path of water molecules within the time frame of observation as is seen in cellular tumors [23]. Loss of cell membrane integrity and reduction in tumor cell density results in an increased ADC measured by DW-MRI as water moves more freely through the tissue, while ADC is lower in more cellular aggressive tumors [24]. By compiling the ADC values derived from each voxel, a parametric map can be generated on which regions of low signal intensity signify restricted diffusion (the reverse of their appearance on source DW-MRI images). ADC metrics can also be represented using histogram analysis to describe mean, median, skewness, and kurtosis of their distribution within a region-of-interest.

Other considerations when implementing DW-MRI are fat suppression (to avoid contamination from fat signals) and adequate anatomical coverage. Optimal fat suppression methods vary as fat suppression efficiencies vary greatly between field-strengths and scanner platforms. Combinations of spectrally-selective and Inversion Recovery fat suppression methods are often required to achieve acceptable DW-MRI images at 3.0-T [25]. Typical imaging protocols used at 1.5 T and at 3 T are given in Table 1. Coverage when imaging HGSOC must extend from the pelvic floor to the hemidiaphragms so requires use of pelvic and torso coils. While this is not problematic in healthy subjects, in patients with distended abdomens who may be breathless because of chest disease, this often represents a limiting factor and a disadvantage compared to CT. As morphological imaging is essential for interpretation, slice positions, field of view, slice thicknesses, and reconstruction matrices of the anatomical images should be matched to the DW-MRI images in order to allow the images to be fused.

## 3. DW-MRI for Differentiating Benign from Malignant Adnexal Masses

Ovarian masses characteristically comprise cystic and solid components. Where cystic components predominate, benign or malignant likelihood is assessed on the morphological features of the cyst (wall thickening, nodules, and septations) on T2-W imaging [27,28]. Whilst the likely benign nature of purely cystic masses (serous and mucinous cystadenomas, which may be uni- or multilocular) is almost certain, papillary projections make accurate assessment challenging. They can be confused for clots, sedimentations, or mucus accumulation. Where solid, measurable components are present, the mean ADC value of solid components in benign ovarian masses has been reported as statistically significantly higher (1.49 ± 0.30 × 10^−3^ mm^2^/s) compared to those in malignant masses (0.95 ± 0.13 × 10^−3^ mm^2^/s) [29]. A recent small study showed that a cut-off ADC value of 0.93 × 10^−3^ mm^2^/s gave a 5.59 times higher risk for malignancy [30]. Although alternatives to mean ADC values have been investigated, parameters such as ADC entropy (a statistical measure representing the irregularity of pixel distribution to reflect tissue heterogeneity) do not outperform reader experience for differentiating benign from malignant masses [31] and are not currently used.

Fibromas of the ovary, which account for approximately 3% of ovarian tumors, have very low ADC values [32] and result in overlap with malignant tumors. Other solid ovarian tumors with collagen-producing fibroblastic cells and a dense network of collagen fibers in the extracellular matrix [33], as seen in benign sex cord-stromal tumors, Brenner tumors, and cystadenofibromas, also return low ADC values. These tumors contribute to the heterogeneity for sensitivity of ADC in identifying malignancy observed in many studies. One study reported ADC for all benign tumors of 0.951 ± 0.625 × 10^−3^ mm^2^/s compared to 0.825 ± 0.129 × 10^−3^ mm^2^/s for malignant sex-cord stromal tumors. Excluding fibromas resulted in improved differentiation (ADC = 1.343 ± 0.5828 × 10^−3^ mm^2^/s) [32]. Cystic degeneration of fibromas, as described in >50% of these tumors in a postmenopausal group [34], serves to increase their ADC and, thus, improves differentiation from malignant lesions. In a meta-analysis of 10 studies, where evaluation of cystic components and morphologically benign appearing tumors, as defined on T2-W images, were excluded, the diagnostic performance of quantitative ADC values for predicting malignancy reached a pooled sensitivity and specificity of 0.91 (95% confidence interval [CI] 0.88–0.93) and 0.91 (95% CI 0.87–0.94), respectively, and an AUC of 0.96 [27], supporting quantitative ADC values as a potential diagnostic marker in distinguishing benign vs malignant ovarian lesions [35]. Taken out of the context of morphological imaging, ADC retains some diagnostic potential, but its use is not advocated in this way. A study which included endometriomas, dermoids, hemorrhagic corpus luteum cysts, serous and endometroid cystadenocarcinomas, and ovarian metastases, and which included all cystic and solid components of tumor on a single axial slice, showed that ADC and kurtosis-derived ADC were lower and apparent kurtosis coefficient (K_app_) was significantly higher in malignant compared with benign ovarian lesions [36]. However, lesion classification by this method (55% of benign lesions classified correctly) is insufficient to make it a viable clinical tool, particularly where calcified elements are a key component of the differential diagnosis [37] and morphological imaging must be utilized in tandem with DW-MRI.

**Figure 2:**
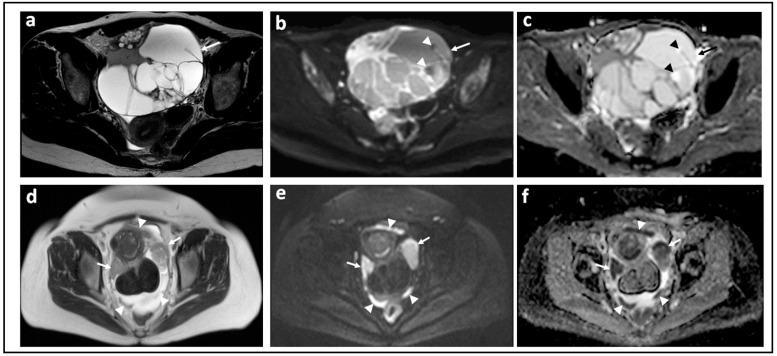
Borderline versus malignant ovarian tumor: T2-W image (**a**) and corresponding b = 1000 s/mm^2^ DW-MRI (**b**) and ADC map (**c**) through the mid-pelvis in a patient with a mucinous borderline tumor of the left ovary. There is a large multiloculated cystic mass with no significant solid components (arrows). Two small foci of low signal in (**b**) show no evidence of diffusion restriction in c (arrowheads). In comparison, the T2-W image (**d**), b = 900 s/mm^2^ DW-MRI (**e**), and ADC map (**f**) in a patient with invasive high grade serous ovarian cancer show bilateral solid irregular ovarian masses (arrows) and linear peritoneal metastases [arrowheads], all of which show marked diffusion restriction.

Borderline ovarian tumors represent 15–20% of primary ovarian neoplasms [38]. Despite their favorable prognosis, their surgical management (hysterectomy, bilateral salpingo-oophorectomy, and omentectomy), entails loss of fertility. Preoperative recognition would enable strategies to retain fertility, e.g., cystectomies, ovarian stroma preservation, and unilateral salpingo-oophorectomy, therefore, DW-MRI has been explored as a potential clinical tool (Figure 2). Various authors have investigated sophisticated ways to assess tiny solid components [39,40,41] including whole solid tumor histogram analysis models, diffusion kurtosis modelling, intravoxel incoherent motion models, or different non-Gaussian diffusion models. He et al. excluded tumors with solid components smaller than 10 mm and carefully avoided hemorrhagic, necrotic, and cystic components and reported that diffusion kurtosis metrics (DKp10) yielded a sensitivity of 86% and an accuracy of 87% at a cut-off ADC value of 1.406 × 10^−3^ mm^2^/s [42]. A meta-analysis of six studies by Pi et al. showed that for differentiating borderline from malignant tumors, the pooled sensitivity and specificity values were 0.89 (95% confidence interval 0.82–0.94) and 0.79 (95% confidence interval 0.73–0.84), and the AUC was 0.91 [27]. However, ADC thresholds cited across studies remain variable. Future use of ADC metrics for differentiation of borderline from invasive malignancy must include clinical, biochemical, and morphological features in an algorithm in order to be clinically useful.

It is not possible to utilize ADC per se to characterize malignant ovarian masses. Like HGSOC, rare malignant solid tumors, such as dysgerminomas, also have characteristically low ADC (0.81 × 10^−3^ mm^2^/s [43]; 0.830 ± 0.154 × 10^−3^ mm^2^/s) [44], as do the solid components of carcinoid tumors [45], with little or no information on other conditions such as malignant struma ovarii or yolk sac tumor. Nevertheless, an inverse relationship between tumor cellularity and ADC may be indicative of tumor aggressiveness: the mean ADC value of clear cell carcinomas has been shown to be higher due to their low cellularity compared to their more cellular counterparts, endometroid, and serous cell cancers [46]. Another study comparing high grade serous [n = 107] vs. low grade serous carcinoma [n = 19] that used the whole solid tumor volume region of interest showed that mean and centile values of ADC were significantly lower in high grade tumors and that skewness of the ADC distribution was higher [41]. Low ADC in a small prospective study of 40 patients also correlated with poor outcome [26], making it a potentially useful biomarker in patient management. However, thresholds for poor outcome remain to be established in large trials [47].

## 4. The Role of DW-MRI in Disease Staging and Predicting Resectability

The stage of ovarian cancer (extent of tumoral spread at diagnosis), typically established by imaging and at surgery predicts patient outcome and dictates the timing of debulking surgery in relation to chemotherapy. The International Federation of Gynecology and Obstetrics (FIGO) staging system (Table 2) [47,48] or one that follows the TNM classification and is approved by the American Joint Committee on Cancer [49] are both used.

**Figure 3:**
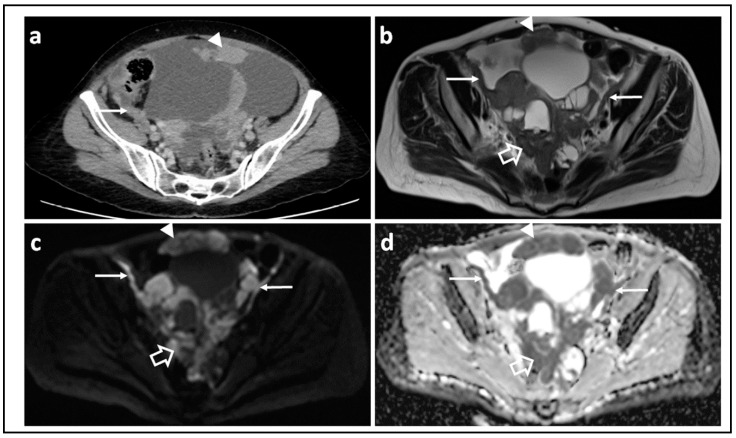
Assessing disease extent using CT versus MRI: CT scan (**a**) T2-W image (**b**) and corresponding b = 1050 s/mm^2^ DW-MRI (**c**) and ADC map (**d**) through the mid-pelvis in a patient with high grade serous ovarian cancer before treatment. The peritoneal (arrow) and omental disease (arrowhead) is evident in (**a**), but the extent of peritoneal and serosal disease encasing the sigmoid colon is more striking on MRI (open arrows) where it appears as high signal within the pelvis in (**c**). Marked diffusion restriction is confirmed in (**d**).

CT of the abdomen and pelvis extended to the chest is the first line imaging modality for staging ovarian cancer as recommended by the European Society of Uro-Genital Radiology (ESUR) [50]. Its excellent spatial resolution, speed of acquisition, wide availability, and cost effectiveness makes it a suitable ‘work horse’ for imaging ovarian cancer patients. Sensitivity of CT in detecting peritoneal metastasis has been reported to range from 64% to 93% with specificity of 92–100% [51]. However, the sensitivity been shown to substantially decrease for peritoneal metastases measuring <1 cm [52] and the sensitivity of CT at certain anatomic regions such as the right subdiaphragmatic space, small bowel mesentery and serosa is limited by low contrast between malignant deposits and adjacent structures. Sensitivity values as low as 22% have been reported in these areas [51].The superiority of MRI compared to CT with regards to soft-tissue contrast resolution results in superior performance: sensitivity and specificity for predicting suboptimal debulking were 0.91 (95% CI, 0.59–1.0) and 0.97 (95% CI, 0.87–1.0), respectively, compared to 0.50 (95% CI, 0.12–0.88) and 1.0 (95% CI, 0.91–1.0), respectively, for CT [53], although not all reports reflect this improved performance [54]. Further improvements in sensitivity and specificity for depicting peritoneal metastases have been shown by the addition of DWI-MRI to T2-W MRI [55,56] (Figure 3). In 255 sites of histologically proven peritoneal disease at surgical excision, the combination of DW-MRI and T2-W MRI for two independent observers was most sensitive and accurate (sensitivity, 0.90 and 0.84, respectively; accuracy, 0.91 and 0.88, respectively) compared with DW-MRI alone (0.71 and 0.71, respectively; accuracy, 0.81 and 0.81, respectively), or conventional MRI alone (sensitivity, 0.73 and 0.52, respectively; accuracy, 0.81 and 0.72, respectively) [55].

A whole-body (WB) DW-MRI technique has recently been advocated for staging and assessing operability [57]. Compared with CT and ^18^FDG-PET/CT, WB-DWI/MRI allowed more accurate tumor characterization and detection of peritoneal, mesenteric, and serosal metastases in a small cohort of 32 patients [58]. For the detection of thoracic lymphadenopathy, qualitative assessment of WB-DWI/MRI significantly improved detection compared with CT, although performance compared with ^18^FDG-PET/CT was similar [58]. For two independent readers, interobserver agreement was moderate to perfect (0.58–1.0) depending on anatomic site [58]. The superiority of WB-MRI was subsequently confirmed in a more recent study with a larger patient population [59]. Moreover, a prospective study in ovarian cancer comparing the DW-MRI with surgical peritoneal cancer index (PCI, frequently used in digestive carcinomatosis patients considered for cytoreduction) in patients undergoing primary or secondary cytoreduction showed a significant correlation between MRI and surgical scores that was better than that reported for ^18^FDG-PET/CT or CT [56]. Interclass correlation coefficients of DW-MRI with surgical staging using the PCI have been shown to be >0.85 for independent observers in a small single center study [60]. Nevertheless, despite this emerging data where DW-MRI is increasingly recognized as superior to CT for staging and assessing operability, its definitive clinical use for predicting resectability remains to be proven. This is currently being assessed in a prospective multicenter trial in the UK whose report will indicate under what circumstances DW-MRI should be used for optimal patient benefit.

## 5. DW-MRI for Longitudinal Follow-Up and to Assess Response

**Figure 4:**
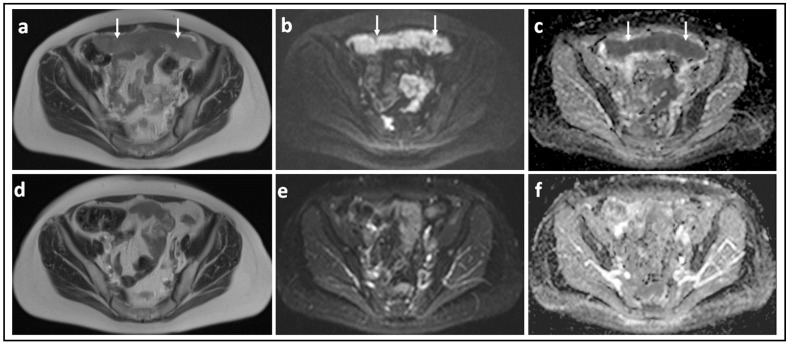
Omental cake showing marked response to chemotherapy: T2-W image (**a**) and corresponding b = 900 s/mm^2^ DW-MRI (**b**) and ADC map (**c**) through the mid-pelvis in a patient with high grade serous ovarian cancer before treatment shows a large omental cake in the anterior pelvis. It is recognized by its homogenous solid appearance and marked diffusion restriction (arrows). Corresponding slices of T2-W (**d**), b = 900 s/mm^2^ DW-MRI (**e**) and ADC map (**f**) after three cycles of platinum-based chemotherapy illustrate that the omental cake is no longer identifiable.

**Figure 5:**
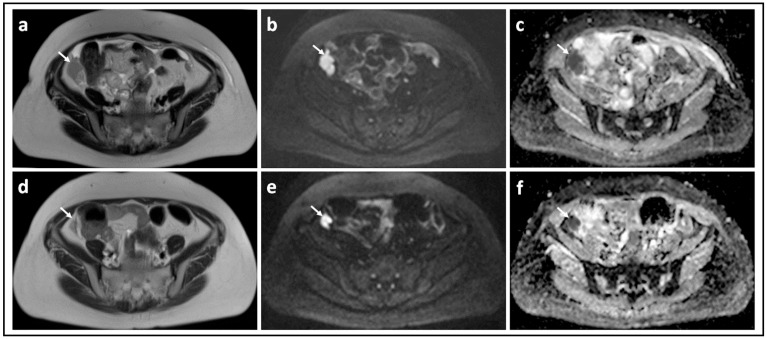
Peritoneal metastasis showing poor response to chemotherapy: T2-W image (**a**) and corresponding b = 900 s/mm^2^ DW-MRI (**b**) and ADC map (**c**) through the upper pelvis in a patient with high grade serous ovarian cancer before treatment shows an irregular right-sided peritoneal metastasis. It is recognized by its homogenous solid appearance and marked diffusion restriction [arrows]. Corresponding slices of T2-W (**d**), b = 900 s/mm^2^ DW-MRI (**e**) and ADC map (**f**) after three cycles of platinum-based chemotherapy illustrate that there has been marginal reduction in the size of this lesion.

DW-MRI may be used qualitatively for evaluating clinical response to NACT (Figure 4 and Figure 5). Qualitative assessment overcomes the limitations of assessing low volume disease where the size of the implants is less than 1 cm, particularly post NACT, and where the sensitivity of CT is reduced to 7–50% [52,61]. However, although the high contrast-to-noise ratio of DW-MRI provides enhanced detectability, both pre and post therapy, it varies with anatomic location. Malignant deposits on the visceral peritoneum are more conspicuous on DW-MRI because of signal suppression from surrounding ascites, bowel contents, and fat. One study showed an incremental value over contrast enhanced MRI in mesentery, serosa of the small bowel and colon, and surface of the uterus and bladder [55], making visual response assessment in these locations with DW-MRI potentially valuable. Small single-center retrospective studies have also indicated that qualitative assessment of DW-MRI is superior to detecting recurrence in patients with suspected recurrence compared to CT (accuracy 94% vs. 78%), particularly at sites such as the mesenteric root and small bowel and colon serosa [62].

Use of the quantitative biomarker, the ADC, derived from DW-MRI for response assessment requires more rigorous technical attention during image acquisition and analysis. In order to be reliable enough for clinical decision-making, the derived ADC needs to be both accurate (close to the reference standard or ground-truth it represents) and precise (both repeatable and reproducible). Repeatability assesses the “closeness of the agreement between the results of successive measurements of the same measurand carried out under the same conditions of measurement”, while reproducibility assess the “closeness of the agreement between the results of measurements of the same measurand carried out under changed conditions of measurement”, such as a different MRI scanner [63]. Thus, the repeatability of ADC estimates determines the ability of the technique to detect treatment-induced changes and determine the size of post-treatment changes that can be detected in individual patients. It also influences the number of patients required for clinical trials. Repeatability is observer dependent because of variations in lesion perception and segmentation. While limits of agreement of ADC measurements can be constrained to within 10% in a quality assured and controlled multicenter setting [64], a small study of 10 women at baseline and after one and four cycles of chemotherapy showed that intra/inter-observer reproducibilities declined through chemotherapy (intraclass correlation coefficients 1 at baseline vs. 0.643 after four cycles of chemotherapy) [65]. This is something to be aware of as lesions shrink and become less well-defined through treatment. It is unlikely that ADC will find a decision-making role in healthcare until vendors incorporate adequate ADC reliability into scanner maintenance (just as RECIST relies on dimensional accuracy verified by scanner maintenance).

Quantifiable treatment-induced changes measured by ADC may occur earlier than conventional morphologic alterations [66,67]. The changes may well depend not only on the class of therapeutic agent used, but also on the anatomic location of the implant. Significant ADC changes have been documented in pre-clinical xenograft models of ovarian tumor after 4 weeks of treatment with single-agent paclitaxel and combination carboplatin/paclitaxel treatment regimens; the maximum ADC was shown to be a good indicator of treatment-induced cell death and changes in the extracellular matrix [68]. In a clinical setting, where implants occur at morphologically different anatomic locations, e.g., ovary, peritoneum, omentum, and where the mean ADC for peritoneal metastases is significantly lower than for ovarian and omental sites [69], differential site-dependent increases in ADC in response to treatment are to be expected. In the prospective multicenter study DISCOVAR, where ADCs were obtained in 47 subjects who subsequently were treated with delayed primary debulking surgery after three or four cycles of NACT, tumor volume reduced at all sites after NACT and ADC increased between pre- and post-NAC measurements. Post-NACT, ADC correlated negatively with tumor cell fraction and positively with percentage necrosis. Significant correlations were driven by peritoneal lesions indicating that following NACT, the ADC increases differentially at disease sites despite similar tumor shrinkage, making its utility site-specific [70]. The value of quantitative DW-MRI in predicting residual disease also merits further interrogation. In a preliminary single center study of 49 patients, tumor ADC normalized to skeletal muscle showed a significant association with the presence of residual tumor at surgery [71].

An analysis of the distribution of ADC values within a lesion provides another approach to assessing disease response. An early study using histogram analysis of ADC values showed that in responders, all centile ADC values increased significantly after the first and third cycle, while skew and kurtosis decreased significantly after the third cycle, but that in non-responders, no parameter changed significantly [72]. A follow-on multicenter study showed that even in relapsed disease where patients were re-challenged with platinum, ADC increased by 47% after one cycle and by 53% after three cycles of chemotherapy. Moreover, the percentage change from baseline differed between responders and non-responders after three cycles; an ADC increase after one cycle was associated with longer PFS in relapsed disease (adjusted hazard ratio, 0.86; 95% CI: 0.75, 0.98; *p* = 0.03) [64]. A histogram analysis approach may also be used to investigate the biologic heterogeneity of tumor by classifying domains of different diffusivity [73,74].

Quantitative diffusion-weighted imaging also may be useful for characterizing postsurgical findings, with high ADC values being more likely to represent areas of edema or inflammation and low ADC values being suggestive of the presence of active tumor cells [75], though the clinical utility in this situation depends on individual patient management.

## 6. Introducing DW-MRI into Clinical Trials?

As with imaging in all clinical trials, when introducing DW-MRI into clinical trials of HGSOC, standardization of all stages of image acquisition, manipulation, analysis, and quantitative assessment is mandatory. Pre-specified imaging protocols, and variations in these must be recorded: poor protocol compliance or inaccurate recording has the potential to alter the images and, therefore, the measured outputs. This is essential because clinical trials are key in informing changes in clinical practice. Rigorous trial conduct and an audit trail with absolute transparency are, therefore, mandatory elements for a successful and reliable trial.

Standard protocols for DW-MRI in ovarian cancer have been developed based on initial measurements using test-objects and volunteers to produce images of an acceptable quality for use in multicenter clinical trials [25]. Standardized fields of view, matrices, numbers of slices, slice thicknesses, number of stations, *b*-values, and sequence parameters across scanner platforms need to be based on assessments of scanner capability in order to achieve the best possible signal-to-noise ratio in all cases. Some parameters can be fixed across all platforms, for example, *b*-values. Three *b*-values, in addition to b = 0 s/mm^2^, are ideal for ADC quantification and allow non-linear models to be used; advanced diffusion sequences (ten *b*-values) acquired at a single station through the largest lesion may be used to enable more sophisticated diffusion modelling [22].

Following protocol development, protocol optimization requires site visits to assess the capabilities of all scanners in the study and refinement of the protocol based on the results from all sites. Test-objects must be used to assess fat suppression, geometric distortion, ghosting, contrast-to-noise, presence of artefacts, and accuracy of ADC estimates between scanners at different sites [76]. Volunteer imaging, though not mandatory, ensures that images from the finalized protocol meet the requirements of all radiologists involved. Morphological components of the imaging in order to cover the whole abdomen and pelvis may not require the same rigor for standardization across scanner platforms if they are not used for quantitation, but need to be matched to the DW-MRI in terms of voxel dimensions, field of view, and slice thickness to enable overlaying of DW-MRI for lesion localization.

The clinicaltrials.gov website currently lists only eight trials in ovarian cancer that utilize DW-MRI (Table 3). Only one of these (the DISCOVAR trial in the UK) is multicenter and quantitative. The other seven trials are predominantly single center with between 10 and 50 participants. Three describe the quantitative use of DW-MRI with derivation of ADC metrics and five utilize qualitative assessments or observer scoring of DWI images. The DISCOVAR trial involved quality assurance and quality control procedures across six sites in the UK. Regular site visits from the trial team, data inspection, and inclusion of investigators at all sites throughout the trial period ensured compliance with trial protocol, good recruitment, and follow-up. In addition, reproducibility data was possible as there was no consideration of additional radiation dose or extrinsic contrast administration with diffusion-weighted MRI. Establishing that under these conditions, the coefficient of variation of ADC was 3.1% [22], enables thresholds to be set for ADC as a response biomarker in a multicenter setting, so that incorporation of quantitative DWI-MRI is possible in future trials of abdomino-pelvic tumors.

## 7. Conclusions

DW-MRI provides qualitative and quantitative evaluation of disease in ovarian cancer and must be implemented with an understanding of its strengths and limitations (Table 4). A technique with a minimum of three *b*-values allows modelling of the signal decay to derive a quantitative biomarker-the ADC. A low *b*-value of 100 s/mm^2^ ensures that perfusional elements are excluded, while a high *b*-value of 1000 s/mm^2^ is ideal to avoid signal decay that lies within the image noise floor. Characterization of adnexal masses, however, based on quantitative ADC assessments alone, is unreliable; here morphological imaging and assessment of cystic and solid components is the diagnostic key. However, for malignant adnexal masses, ADC is inversely correlated with cellularity and is indicative of poor prognostic outcome.

Qualitative assessment of abdomino-pelvic DW-MRI is increasingly recognized as valuable in ovarian cancer staging. Its role in comparison to currently performed CT staging will emerge from direct comparison of these modalities in on-going multi-center trials. The ADC may be incorporated into clinical trials of ovarian cancer as a response biomarker but requires careful quality assurance and quality control procedures during the conduct of the trial to minimize measurement variability and ensure reproducibility. The ADC has potential to indicate response in individual peritoneal lesions after one cycle of NACT and prior to size changes being evident. In future, it’s potential for guiding tailored management of chemotherapy and targeted agents not only within clinical trial protocols but also in clinical routine will become established. 

## Figures and Tables

**Table 1:** Pulse sequence parameters used at 1.5 T and 3.0 T.

Parameter	1.5 T ^a^	3.0 T
**Receive coil**	anterior body matrix and posterior spine matrix; 32 channel body array	body coil [Sense-XL-Torso] ^b^; 8 channel cardiac array ^c^
**Slice orientation**	axial	axial ^b,c^
**Breathing**	Free breathing	Breath-hold [upper abdomen] or free-breathing [pelvis] ^b^; free-breathing ^c^
**Sequences**	Single shot EPI	Single shot EPI ^b,c^
**Averages**	4	
**FOV** **/mm [read] × mm [phase]**	380 × 332	340 × 340
**Acquired matrix**	128	160 ^c^
**Reconstructed matrix**	256	160 ^c^
**Acquired pixel size/mm × mm**	3 × 3	1.8 × 1.8 ^b^
**Slice thickness/mm**	6	5 [0.5 gap] ^b^; 5 [1 mm gap] ^c^
**No. of slices**	26	48–56 ^b^
**Parallel imaging**	GRAPPA [reduction factor 2; 36 ACS lines]; ASSET reduction factor 2	SENSE factor 2 ^b,c^
**Phase encode direction**	AP	not available
**Receive bandwidth**	1776 Hz/pixel; ±125 kHz [1953 Hz/pixel]	250 kHz ^c^
**TR/ms**	8000	2600 ^c^
**TE/ms**	75; 81	71.5 ^c^
**Fat suppression**	SPAIR; water selective excitation	STIR ^b^
**Diffusion gradient scheme**	Bipolar; DSE	not available
**Diffusion encoding scheme**	Three-scan trace; ALL	not available
**Diffusion weightings [*b*-values] for full volume coverage/s mm^−2^**	0, 100, 500, 900	0, 300, 600 ^b^
**Diffusion weightings [*b*-values] for additional station/s mm^−2^**	0, 50, 100, 150, 200, 250, 300, 500, 700, 900	0, 30, 50, 100, 150, 200, 400, 600, 800, 1000, 1500 ^c^

^a^ = Winfield et al. [25]; ^b^ = Lindgren et al. [26]; ^c^ = Wang et al. [21]. DSE = Dual Spin-Echo; EPI = Echo-Planar Imaging; GRAPPA = GeneRalized Autocalibrating Partial Parallel Acquisition; SENSE = SENSitivitity Encoding; SPAIR = SPectral Adiabatic Inversion Recovery; STIR = Short Tau Inversion Recovery.

**Table 2:** FIGO staging of carcinoma of the ovary, fallopian tubes and peritoneum [48].

Stage 1 Tumor Confined to Ovaries or Fallopian Tube[s]	T1-N0-M0
IA: tumor limited to one ovary [capsule intact] or fallopian tube; no tumor on ovarian or fallopian tube surface; no malignant cells in the ascites or peritoneal washings	T1a-N0-M0
IB: tumor limited to both ovaries [capsules intact] or fallopian tubes; no tumor on ovarian or fallopian tube surface; no malignant cells in the ascites or peritoneal washings	T1b-N0-M0
IC: tumor limited to one or both ovaries or fallopian tubes, with any of the following: IC1: surgical spill IC2: capsule ruptured before surgery or tumor on ovarian or fallopian tube surface IC3: malignant cells in the ascites or peritoneal washings	T1c1-N0-M0 T1c2-N0-M0 T1c3-N0-M0
**Stage II. Tumor involves one or both ovaries or fallopian tubes with pelvic extension [below pelvic brim] or primary peritoneal cancer**	**T2-N0-M0**
IIA: extension and/or implants on uterus and/or fallopian tubes and/or ovaries	T2a-N0-M0
IIB: extension to other pelvic intraperitoneal tissues	T2b-N0-M0
**Stage III. Tumor involves one or both ovaries or fallopian tubes, or primary peritoneal cancer, with cytologically or histologically confirmed spread to the peritoneum outside the pelvis and/or metastasis to the retroperitoneal lymph nodes**	
IIIA1: positive retroperitoneal lymph nodes only [cytologically or histologically proven]: IIIA1[i] Metastasis up to 10 mm in greatest dimension IIIA1[ii] Metastasis more than 10 mm in greatest dimension	T1/T2-N1-M0
IIIA2: microscopic extrapelvic [above the pelvic brim] peritoneal involvement with or without positive retroperitoneal lymph node	T3a2-N0/N1-M0
IIIB: macroscopic peritoneal metastasis beyond the pelvis up to 2 cm in greatest dimension, with or without metastasis to the retroperitoneal lymph nodes	T3b-N0/N1-M0
IIIC: macroscopic peritoneal metastasis beyond the pelvis more than 2 cm in greatest dimension, with or without metastasis to the retroperitoneal lymph nodes [includes extension of tumor to capsule of liver and spleen without parenchymal involvement of either organ]	T3c-N0/N1-M0
**Stage IV. Distant metastasis excluding peritoneal metastases**	
Stage IVA: pleural effusion with positive cytology	Any T, any N, M1a
Stage IVB: parenchymal metastases and metastases to extra-abdominal organs [including inguinal lymph nodes and lymph nodes outside of the abdominal cavity]	Any T, any N, M1b

**Table 3:** Clinical trials incorporating DW-MRI listed on clinicaltrials.gov.

Title	Conditions	Interventions	No. Participants Planned	Primary Outcome Measure	DWI Assessments	Single/ Multicentre	Location
Imaging Study in Advanced Ovarian Cancer	Ovarian cancer	Diagnostic Test: Ultrasound, CT and WBDWI/MR	400	Preoperative identification of patients with ovarian/tubal cancer in whom optimal debulking (R0/R1) can not be achieved by US and CT scan	Qualitative	Single	Gynecologic Oncology Center in Prague, Prague, Czechia
Clinical Impact of Dedicated MR Staging of Ovarian Cancer	Ovarian cancer	Other: MRI	270	Diagnostic performance of DW-MRI to predict a complete cytoreductive surgery	Qualitative	Single	NKI-AVL, Amsterdam, Netherlands
Value of MRI in the Characterization of Ovarian Masses Unable to Classify With Ultrasound Using the International Ovarian Tumor Analysis (IOTA) Simple Rules	Patients With a Sonographically Unclassifiable Adnexal Mass Using the IOTA Simple Rules	Other: Diffusion/Perfusion-weighted Magnetic Resonance Imaging	250	The sensitivity and specificity of the ADNEXMR SCORING system in classifying adnexal masses as malignant or benign using MRI with diffusion- and perfusion-weighted sequences in masses unclassified by the IOTA Simple Rules. Gold standard is histopathology diagnosis within 120 days after ultrasound examination.	Radiologist scoring	Single	University Hospitals Leuven, Leuven, Belgium
Benchmarking Intra-tumor Heterogeneity In Ovarian Cancer: Linking In-vivo Imaging Phenotypes With Histology And Genomics	Ovarian cancer	Procedure: ^18^FDG-PET/CT Scan Procedure: MRI	26	Genomic markers of spatial heterogeneity by evaluating spatially explicit phenotypic clusters based on a combination of perfusion, diffusion and metabolic tumor profiles (maps) in both ovarian tumors and metastatic peritoneal/omental implants of patients with HGSOC undergoing primary debulking surgery.	Quantitative	Single	Memorial Sloan Kettering West Harrison, Harrison, New York, United States Memorial Sloan Kettering Cancer Center, New York, New York, United States
Whole-body Diffusion MRI for Staging, Response Prediction and Detecting Tumor Recurrence in Patients With Ovarian Cancer	Ovarian carcinoma	Other: Whole body DW-MRI	350	WB-DW-MRI for tumor characterization and staging at primary diagnosis and response prediction to neoadjuvant chemotherapy	Qualitative	Single	University Hospitals UZ Leuven, Gasthuisberg, Leuven, Belgium
Diffusion-weighted Imaging Study in Cancer of the Ovary	Ovarian Cancer Peritoneal Metastases	DW-MRI	134	To assess the reproducibility of quantitative diffusion-weighted magnetic resonane imaging (DW-MRI) for visualising peritoneal metastases in a multi-centre setting and biologically validate the measurements by correlating scan data (ADC change) following chemotherapy with histology of the tumor (amount of cell death) at surgery	Quantitative	Multicentre	Addenbrookes Hospital, Cambridge University Hospitals NHS Foundation Trust, Cambridge, Cambridgeshire, United Kingdom Queen Elizabeth Hospital, Newcastle, Gateshead, United Kingdom Mount Vernon Cancer Centre, Northwood, Middlesex, United Kingdom The Institute of Cancer Research and Royal Marsden NHS Foundation Trust, Sutton, Surrey, United Kingdom Singleton Hospital, Swansea, Wales, United Kingdom Imperial College Healthcare NHS Trust, London, United Kingdom
Whole Body Diffusion MRI for Non-invasive Lesion Detection and Therapy Follow-up: Study With Patients With Ovarian Cancer and Peritoneal Metastasis	Ovarian Cancer Peritoneal Metastases	Procedure: intravenous contrast administration	50	To evaluate which of the two treatments (primary debulking surgery followed chemotherapy versus platinum-based neoadjuvant chemotherapy followed by interval debulking surgery, followed in turn by chemotherapy) is the best option for a particular type of patient.	Qualitative	Single	University Hospital Gasthuisberg, Leuven, Belgium
Evaluation of Response to the Neoadjuvant Chemotherapy for Advanced Ovarian Cancer by Multimodal Functional Imaging	Ovarian carcinoma	Procedure: ^18^FDG-PET/CT and DW-MRI before and after 4 cycles of neoadjuvant chemotherapy	11	Inter-rater Reliability of Magnetic Resonance Imaging (MRI) Apparent Diffusion Coefficient (ADC)	Quantitative	Single	Institut Bergonié Bordeaux, Gironde, France

**Table 4:** Summary of pearls and pitfalls of DW-MRI for imaging high grade serous ovarian cancer.

Clinical Need	Pearls	Pitfalls
**Technical performance**	A low *b*-value of 50–100 s/mm^2^ ensures that diffusivity measurements are not contaminated by contributions from microcapillary flow A high *b*-value of 1000 s/mm^2^ is optimal for detecting metastatic HGSOC Fat suppression may require a combination of methods to achieve optimal cancellation of fat signals	Very high *b*-values [>2000 s/mm^2^] mean that signal is at the level of the noise floor Geometric distortion reduces reliability of fusion of DW-MRI with anatomic images
**Detecting and Characterising malignant lesions**	Cellular masses, whether benign or malignant, have low ADCs Morphological imaging with assessment of cystic and solid elements is key for lesion characterization	ADC alone should not be used to differentiate benign from malignant disease
**Staging and Resectability**	In single center studies, WB-MRI with DW-MRI is comparable to FDG-PET for disease staging The utility of DW-MRI for replacing CT as the imaging modality of choice in disease staging remains to be demonstrated in a multicenter trial	Although used qualitatively for staging, meticulous technique is essential to minimize artefacts. WB-MRI is needed to ensure coverage of the thorax, abdomen, and pelvis for complete staging
**Response assessment**	ADC changes indicative of response are anatomic site-specific ADC changes indicative of response are evident after one cycle of platinum-based chemotherapy in relapsed disease when re-challenged with platinum	Lack of knowledge of accuracy and precision of the ADC measurement can lead to incorrect response classification
**Incorporation into clinical trials**	ADC repeatability is achievable in quality controlled clinical trials Current prospective trials utilizing ADC as a response biomarker in HGSOC should make use of site-specific and perilesional response assessments	Non-Standardized ADC measurements obtained without quality assurance and quality control are subject to substantial variation

## Data Availability

Not applicable.
